# Magnitude of observer error using cone beam CT for prostate interfraction motion estimation: effect of reducing scan length or increasing exposure

**DOI:** 10.1259/bjr.20150208

**Published:** 2015-09-03

**Authors:** Helen A McNair, Emma J Harris, Vibeke N Hansen, Karen Thomas, Christopher South, Shaista Hafeez, Robert Huddart, David P Dearnaley

**Affiliations:** ^1^ Royal Marsden NHS Foundation Trust, Sutton, UK; ^2^ Institute of Cancer Research, Sutton, UK; ^3^ Department of Physics, St Luke's Cancer Centre, The Royal County Hospital NHS Foundation Trust, Guilford, Surrey

## Abstract

**Objective:**

Cone beam CT (CBCT) enables soft-tissue registration to planning CT for position verification in radiotherapy. The aim of this study was to determine the interobserver error (IOE) in prostate position verification using a standard CBCT protocol, and the effect of reducing CBCT scan length or increasing exposure, compared with standard imaging protocol.

**Methods:**

CBCT images were acquired using a novel 7 cm length image with standard exposure (1644 mAs) at Fraction 1 (7), standard 12 cm length image (1644 mAs) at Fraction 2 (12) and a 7 cm length image with higher exposure (2632 mAs) at Fraction 3 (7H) on 31 patients receiving radiotherapy for prostate cancer. Eight observers (two clinicians and six radiographers) registered the images. Guidelines and training were provided. The means of the IOEs were compared using a Kruzkal–Wallis test. Levene's test was used to test for differences in the variances of the IOEs and the independent prostate position.

**Results:**

No significant difference was found between the IOEs of each image protocol in any direction. Mean absolute IOE was the greatest in the anteroposterior direction. Standard deviation (SD) of the IOE was the least in the left–right direction for each of the three image protocols. The SD of the IOE was significantly less than the independent prostate motion in the anterior–posterior (AP) direction only (1.8 and 3.0 mm, respectively: *p* = 0.017). IOEs were within 1 SD of the independent prostate motion in 95%, 77% and 96% of the images in the RL, SI and AP direction.

**Conclusion:**

Reducing CBCT scan length and increasing exposure did not have a significant effect on IOEs. To reduce imaging dose, a reduction in CBCT scan length could be considered without increasing the uncertainty in prostate registration. Precision of CBCT verification of prostate radiotherapy is affected by IOE and should be quantified prior to implementation.

**Advances in knowledge:**

This study shows the importance of quantifying the magnitude of IOEs prior to CBCT implementation.

## INTRODUCTION

The use of intraprostatic gold markers has improved the accuracy of radiotherapy treatment to the prostate by providing a surrogate of the prostate position which is visible on kV or MV X-ray imaging.^1,2^ However, information regarding deformation of the prostate and organs at risk is not available on 2D planar imaging. The implementation of in-room CT imaging devices has provided 3D information to quantify target motion, rotation and deformation in addition to movement of organs at risk.^3–5^ This enables online 3D imaging, soft-tissue registration and has the potential to reduce planning target volume (PTV) margins, allowing dose escalation with the aim to improve the therapeutic ratio. Whilst online imaging reduces the uncertainties associated with the prostate position, residual errors will remain. One source of residual error is interobserver error (IOE) which has been shown to be significant, albeit with the majority of the studies exporting the cone beam CT (CBCT) and contouring the prostate on treatment planning systems which is not replicating registration at the treatment console.^5–10^ IOEs gave a standard deviation (SD) of contoured prostate volumes as great as 20% of the average prostate volume (10) and a SD of IOE of >2 mm was found.^5,8^ A common finding in all studies was greater interobserver variation in the SI direction.^5–10^ This is consistent with studies which investigated interobserver on CT images.^11–13^ A few studies have compared CBCT with 2D imaging devices^14,15^ and found that compared with kV imaging of intraprostatic fiducial markers, there were differences in set-up errors of >3 mm in the SI and AP direction^14^ and an additional 1 mm margin was required, when using CBCT without intraprostatic fiducial markers.^15^ Although IOEs were not investigated, it was postulated that these differences were, in part, due to the difficulty in visualizing the prostate on the CBCT images.

The above studies demonstrate that IOEs should be considered when using 3D imaging without gold markers for verification and defining planning treatment volume margins. It may also be appropriate to investigate the methods of reducing IOEs. Currently in our department, CBCT images for prostate verification radiotherapy are acquired using XVI v. 4.5 (Elekta Oncology Systems, Crawley, UK). The imaging options can be selected from a choice of small, medium and large, field of view (FOV) equating to approximately 26, 40 and 52 cm diameter FOV and three field lengths 10, 15 and 20 equating to 12, 17 and 26 cm length at the isocentre. The standard imaging protocol for the prostate at our centre is a 12 cm scan length, 40 cm FOV (M10) and 1644 mAs exposure resulting in a cone beam CT dose index (CTDI) of 27 mGy. All the scans for this study are medium FOV, and we refer to the length by the actual lengths, *i.e.* 12 cm for standard and 7 cm for the test length.

One method to reduce IOEs might be to improve the visualization of the prostate by improving CBCT image quality. We propose that the image quality of our standard CBCT images would be improved if a smaller length of tissue was imaged thereby reducing the amount of scattered radiation which has the benefit of reduced integral dose. We also propose to investigate if reducing image scan length increasing the X-ray exposure improves image quality and reduces IOE.

The aim of this study was to firstly, determine IOE in determining prostate position on CBCT images acquired using standard protocol. Secondly, to investigate if reducing the scan length or increasing the exposure affects the magnitude of IOE. IOEs associated with registering the prostate position on CBCT images to planning CT scans were determined using our standard CBCT image protocol (12 cm and 1644 mAs) and compared to those obtained when using:

(1) a reduced CBCT scan length with the same X-ray exposure (7 cm and 1644 mAs) giving a reduced integral dose compared with the standard protocol. The dose length product is reduced from 324 to 189 mGy*cm.
(2) a reduced CBCT scan length and increased X-ray exposure (7 cm and 2632 mAs : CTDI 43.2 mGy) giving equivalent integral dose to the standard protocol. The dose length product is 302 mGy*cm.

IOEs using CBCT were compared with independent prostate motion and the accuracy of prostate position measurement using automated software match.

## METHODS AND MATERIALS

Patients referred for radical radiotherapy to the prostate and seminal vesicles were recruited for the study, which was approved by the local research and ethics committees. Patients were immobilized using the Combi Fix system (Oncology Systems Ltd, Shropshire, UK) and had been given information sheet detailing instructions regarding maintenance of a comfortable full bladder throughout treatment. Planning CT scans were acquired with 3-mm slice thickness. If patients had an anteroposterior rectal dimension of >4 cm at the time of CT planning then the patient was rescanned. If the rectum was distended due to faeces rather than gas, enemas were prescribed for the repeat scan and during treatment. Patients were treated using a three field forward planned intensity modulated radiotherapy treatment (Pinnacle; Philips) delivered in either 2 Gy and 37 fractions or 3 Gy and 20 fractions.

**Figure 1.**
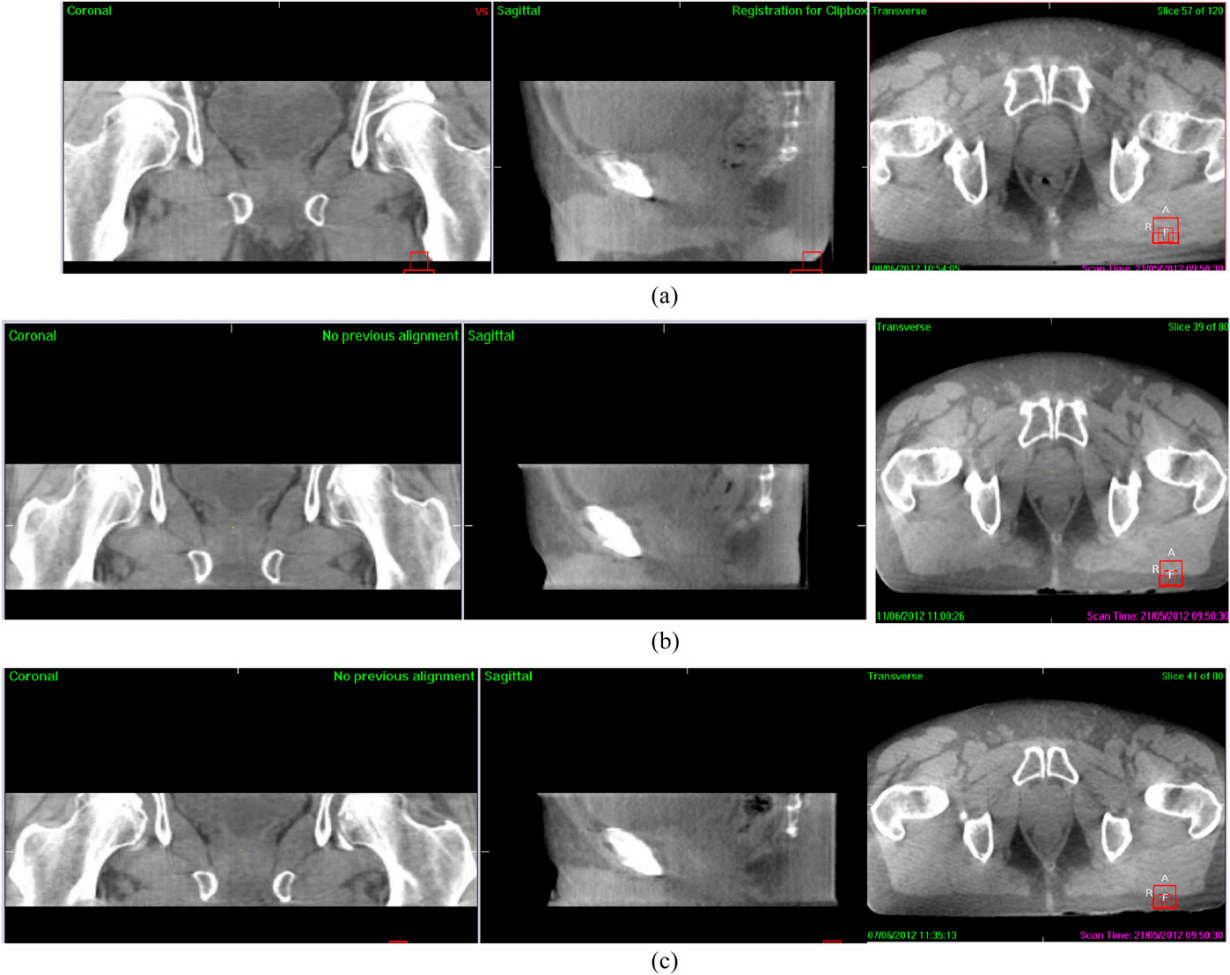
Cone beam CT acquired using (a) standard 13 cm length (M10; 1644 mAs), (b) 7 cm length standard dose (M5; 1644 mAs) and (c) 7 cm length high dose (M5; 2632 mAs).

Prior to treatment delivery, CBCT images were acquired using a novel 7 cm length image with standard exposure (1644 mAs) at Fraction 1 (7), standard 12 cm length image (1644 mAs) at Fraction 2 (12) and a 7 cm length image with higher exposure (2632 mAs) at Fraction 3 (7H) (Figure 1). The remainder of verification images used the standard length and exposure.

Patients were positioned to skin marks and the isocentre position set according to plan set-up. CBCT images were registered to planning CT images retrospectively by eight observers (two clinicians and six radiographers) (Elekta Synergy^®^ XVI v. 4.5; Crawley, UK) using treatment console software (XVI v. 4; Elekta Oncology Systems). Guidelines and training were provided for the observers which included identification and comparison of the prostate in images acquired by MRI, CT and CBCT. Observers were asked to firstly register the images using bony anatomy and then manually adjust, where necessary, using soft tissue. To do this, the observer defined a region of interest which was used by the software to perform automated rigid registration to bony anatomy (chamfer matching). The observer visually checked the registration of the prostate and manually adjusted the registration if necessary to obtain a closer match. Observers recorded prostate position (this was the total set-up error including both patient and prostate displacement) and whether manual adjustment had been performed. In addition, they indicated their confidence in the prostate match on a visual analogue scale of 0–10, where 0 was not confident and 10 was very confident. Patient width, laterally and anteroposterior, and the presence of gas in the images were also recorded to evaluate their effect on IOEs. In addition, one observer registered the images using the automatic dual registration software. This firstly registers the bony anatomy position, followed by the “greyscale” registration (cross correlation) to the soft tissue using an irregular region of interest (mask) defined automatically as the clinical target volume of the prostate plus 0.5 cm margin. It was ensured that there was no bony anatomy included in the mask since this would affect the soft-tissue registration.

### Statistical analysis

For each patient, the average prostate position, across all the observers, provided an estimate of the “gold standard” prostate position (total set-up displacement). To determine independent prostate motion, the bony anatomy positions were subtracted from the prostate position to determine independent prostate motion and to compare that found by other studies.

The IOE for each image was calculated as the SD of the prostate displacement recorded by all eight observers. IOE were tested for normality using a Quantile–Quantile probability plot (Q–Q plot). To compare the means of the IOEs between the three different imaging protocols, a non-parametric unrelated samples test, Kruzkal–Wallis, was used.

Patient size (lateral width and anteroposterior depth) and the presence of gas in the images were also recorded and the effect on IOEs was determined using a Spearman's rank correlation coefficient and Mann–Whitney *U*-test independent samples *U*-test, respectively. The relationship between the visual analogue score and IOE was assessed using Spearman's rank correlation coefficient.

To enable clinical implementation of CBCT soft-tissue imaging, we defined that the uncertainty in the registration should be less than the uncertainty of using bony anatomy. Non-parametric Levene's test was used to test if the variances of the IOEs and the independent prostate position were equal or different.

The automatic greyscale registrations were compared to the observer registrations to determine the magnitude and frequency of manual adjustments.

## RESULTS

### Set-up errors

93 CBCT images were acquired in 31 patients (3 per patient) and the mean (SD) and median (range) of the total set-up interfraction errors (patient and prostate displacement) are shown in Table 1.

**Table 1.** Interfraction set-up displacements

Translational direction	Total set-up errors (patient and prostate)		Independent prostate motion	
	Mean (SD)	Median (range)	Mean (SD)	Median (range)
Right–left (mm)	−0.6 (2.8)	0.7 (5.9–5.6)	−0.2 (0.9)	0 (−5.4 to 0.9)
Superior–Inferior (mm)	1.2 (2.4)	1.0 (3.7–8.7)	0.10 (1.9)	0.2 (−3.7 to 3.9)
Anterior–posterior (mm)	0.5 (3.6)	0.2 (10.7–7.8)	−1.1 (3.0)	0.3 (−12 to 4)

Where left is positive sign, superior is positive sign and anterior is positive sign.

### Interobserver errors

The mean IOE was the greatest in the AP direction for each of the three image protocols but the SD of the IOE was greater in the SI direction (Table 2).

**Table 2.** Mean (standard deviation) of interobserver errors in each direction and image type

Cone beam CT image type	Right–left (mm)	Superior–inferior (mm)	Anterior–posterior (mm)
7 cm standard exposure (*n* = 31)	0.4 (0.5)	1.2 (0.9)	1.6 (0.8)
12 cm standard exposure (*n* = 31)	0.4 (0.3)	1.4 (0.9)	1.6 (0.7)
7 cm high exposure (*n* = 30)	0.5 (0.5)	1.5 (1.0)	1.8 (0.8)

No significant difference was found between the IOEs in any direction between the image protocols; therefore, the IOEs were analysed using all images from hereon in. There was also no significant difference between the two clinicians' results and the six radiographers' results.

The SD of the IOE was significantly less (*p* = 0.017) than the independent prostate motion in the AP direction only (Table 3). IOEs were not significantly different to independent prostate motion in the LR and SI directions.

**Table 3.** Standard deviation of interobserver errors compared to independent prostate motion

Translational direction	SD of interobserver error (mm)	SD independent prostate motion (mm)	*p*-value
Right–left	0.6	1.0	0.82
Superior–inferior	1.6	1.9	0.51
Anterior–posterior	1.8	3.0	0.01

**Figure 2.**
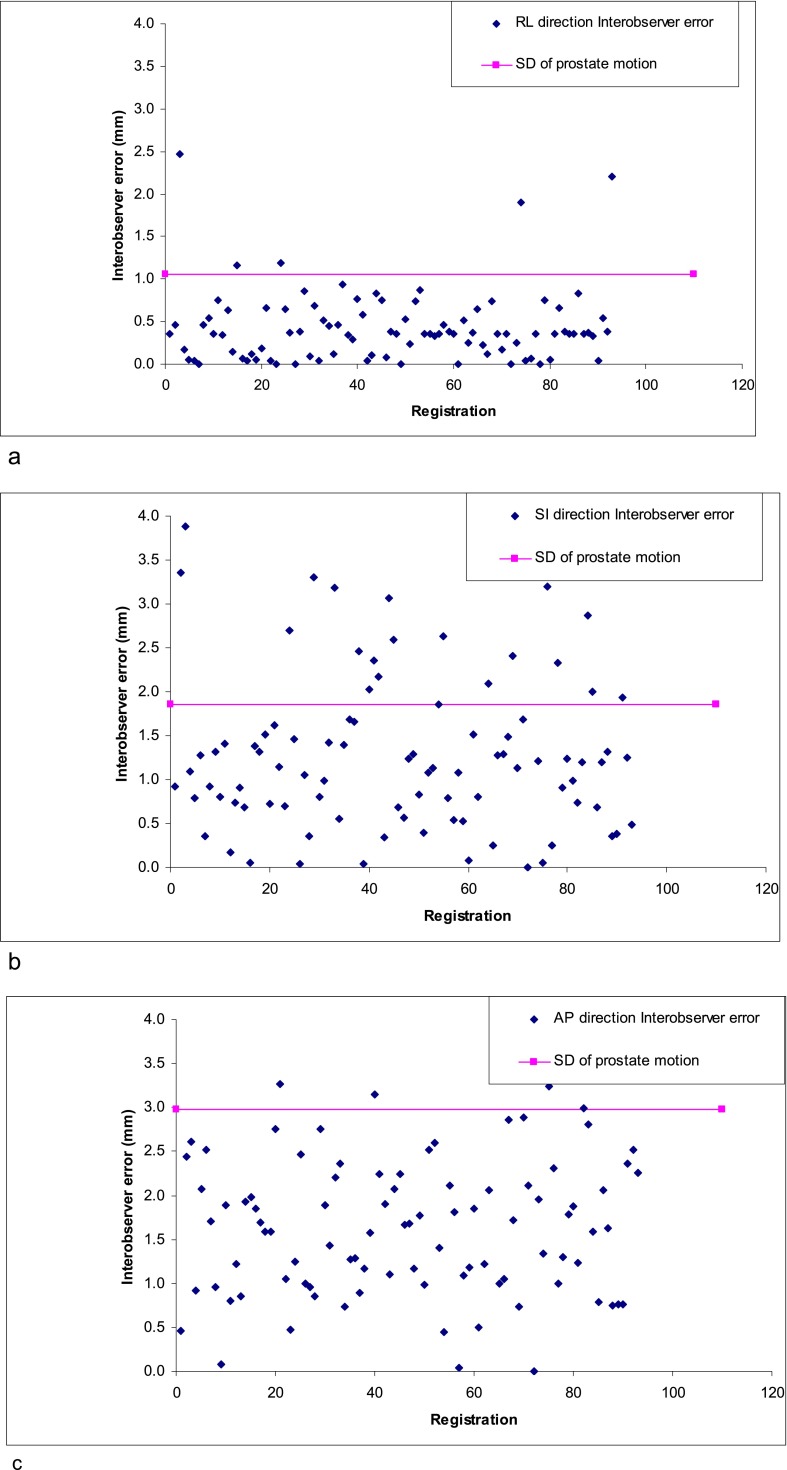
Interobserver errors (mm) as related to SD of prostate motion in, RL (a); SI (b); AP (c); directions.

The IOEs were within 1SD of the independent prostate motion in 95%, 77% and 96% of the images in the RL, SI and AP direction (Figure 2a–c). The IOE was greater when there was gas present in the CBCT image in the RL (*p* = 0.03) and AP direction (*p* = 0.01). There was no significant difference in the SI direction. The IOEs were not affected by patient dimensions.

The confidence score measured with the visual analogue scale was not correlated with the IOE in the RL and SI direction but as confidence increased the IOE decreased (*r* = 0.6; *p* = 0.01) in the AP direction.

### Comparison of observer registrations and automatic registrations

The average interobserver registration correlated strongly (Pearson's product–moment correlation coefficient) with the automatic “greyscale” match in the RL direction (*r* = 0.89; *p* = 0.01 and less strongly in the SI direction (*r* = 0.78; *p* = 0.01) and AP direction (*r* = 0.58; *p* = 0.01).

The difference between the observer registrations and greyscale registrations was >3 mm in 5%, 21% and 15% of the images and >5 mm in 2%, 11% and 8% of the images in the RL, SI and AP directions, respectively. The registration was manually adjusted in 42% of registrations.

## DISCUSSION

Increasing the dose and reducing the length of the CBCT did not have a significant effect on the IOEs. We found the IOEs of CBCT registration with planning CT to be of a magnitude that ought to be considered a component of the residual error. Residual errors can arise from geometrical uncertainties (phantom transfer error), errors with the position measurement and inaccurate couch movement, IOEs associated with CBCT and planning CT registration or patient motion. The SD of residual errors due to mechanical couch movement is reported to be in range of 0.8–1.6 mm.^16,17^ Residual errors >2 mm are generally thought to be due to prostate motion;^17–19^ however, these studies investigated residual error with pre- and post-treatment images, and subsequent investigations have shown that large prostate motions during treatment can be transient.^20^ Our study has shown that IOEs from soft-tissue registrations are similar in magnitude to other sources of residual error and therefore should be quantified and taken into account when calculating clinical target volume (CTV) to PTV margins.

The SD of observer displacements was significantly less than that of the independent prostate motion in one direction only (AP). Comparing the IOEs to a standard 2 mm tolerance used in radiotherapy 1%, 13% and 16% of images in the RL, SI and AP directions, respectively had IOEs of >2 mm. This suggests that centres considering using soft-tissue CBCT match rather than bony anatomy matching, and it is important that IOEs are quantified and compared to expected independent prostate motion. If observer errors are greater, there may be no benefit from using CBCT soft-tissue match.

The greater magnitude of manual moves in the SI direction could be explained by the known difficulty in assessing prostate position in CT scans in this direction.^11–13^ However, the 3-mm slice thickness of the reference CT planning may also contribute to the larger discrepancies on the SI direction. In addition, the difficulty in visualizing the prostate (made worse by blurring because of gas pockets) may also have affected the registrations in all directions.

The lack of “ground truth” of the prostate motion is a weakness of this study; however, the distribution of the set-up errors of both patient and prostate is within expected ranges compared with previously published results.^1,2,21,22^

Reducing the length of the scan and increasing the dose did not improve IOEs, and we suggest that other methods of decreasing IOEs are investigated. Possible solutions to aid image registration include implanting fiducial markers and with the 3D imaging would still provide the additional soft-tissue information regarding organs at risk position and deformation of the target compared with kV or MV planar imaging. Improved training for radiographers may decrease IOE; however, two of these observers were clinicians and the mean of the radiographers and the mean of the clinicians were not significantly different. This agreement suggests that for routine clinical practice prostate matching does not require clinician intervention. Furthermore, routine practice involves two radiographers when checking the final registration which has been shown to make a difference in concordance when selecting PTV for bladder patients.^23^ Reducing the slice thickness of the planning CT scan may decrease the error in the SI direction. However, the findings of this study have highlighted that reducing the scan length did not increase IOEs, and therefore a reduction in scan length may benefit patients, by reducing integral dose with no loss of precision.

## CONCLUSION

Reducing CBCT scan length and increasing exposure did not have a significant effect on IOEs. To reduce imaging dose, a reduction in CBCT scan length could be considered without increasing the uncertainty in prostate registration. Precision of CBCT verification of prostate radiotherapy is affected by IOE which should be quantified prior to implementation.
